# Strengthening of the clinical research capacity for malaria: a shared responsibility

**DOI:** 10.1186/1475-2875-9-S3-S5

**Published:** 2010-12-13

**Authors:** Charles S Mgone

**Affiliations:** 1 European & Developing Countries Clinical Trials Partnership Laan van Nieuw Oost Indië 334, 2593 CE The Hague, Netherlands

## Abstract

Lack of adequate human resource capacity, good governance, sound physical infrastructure and well-functioning systems impede economic growth in low- and middle-income countries. The heavy burden from disease compounds this. To overcome these setbacks a concerted effort needs to be taken. This requires collective effort of all including the public and private sectors from development partners and from low- and medium-income countries themselves. Specific research capacity gaps, such as lack of expertise and infrastructure to engage in upstream research and development of new products, need to be addressed. Special attention should also be given to those with more acute capacity needs and high disease burden, such as communities in conflict-affected regions. Capacity building approaches need to be innovative and responsive to needs and the ever changing scientific landscape. Therefore, for example, as the global community aims to eliminate and eventually eradicate malaria, there should be an appropriately matched effort to strengthen the capacity to meet these challenges.

## Background

Capacity development is the process of enabling individuals or systems to recognize and solve their own problems, make informed choices, define priorities and plan their futures in a sustainable manner. This effort may be targeted to specific individuals, institutions, organizations, communities or entire nations. Similarly it may be targeted to specific tasks and needs or in broad terms at the overall strengthening of systems.

Traditionally, most of the efforts on capacity development in the past have been targeted at individuals, mostly through training. The training may be conducted in the form of workshops, seminars, short- and long-term professional courses or mentoring. However, this may not always be effective since such a trained individual without appropriate supporting structures and enabling environment may not be able to function optimally. A more effective, but not necessarily well-appreciated form of capacity strengthening is at institutional level, whereby efforts are targeted to strengthening policies, governance and programmes. Delivered in this manner, capacity development may not only be aligned to the existing programmes and policies, but also built along the existing supporting structures thus ensuring utilization and sustainability of the strengthened capacity.

A higher level of capacity strengthening is that of national or regional support. Such societal capacity building may involve national policies, governance, laws and regulations. This has in the past generally been very much neglected by many international development partners in favour of projects support. This omission is particularly in the area of research for health, which continues to be neglected, especially within the current favoured approach of general budget support to low- and middle-income countries. Although it may be politically correct to channel aid through general budget support and sector wide approach, quite often such support does not adequately reach health research sector due to many other competing needs. Moreover, there is no evidence to support that such approaches are effective and have a real impact in powering capacities [1].

## Research and research capacity ownership and leadership

Strengthening of the research capacity for malaria must be holistic and viewed as part of the big picture in the development of capacity for research for health, which will in turn contribute to improving community health in general. Primarily, it is the responsibility of every nation to ensure that it can provide the best services including healthcare to its people. This will include having the required capacity in terms of personnel and infrastructure. Unfortunately, this is not often possible in most of the low- and middle-income countries, where due to lack of economic empowerment capacity development mostly depends on foreign aid. Unfortunately, left this way low- and middle-income countries will have no control in addressing to their capacity needs. For capacity development to be effective, the process must be owned and driven by those who need the capacity and tailored to response to their needs. Ownership requires active involvement including participation in decision making on the required capacity, investing on the capacity development processes as well as the utilization and retention of the developed capacity. Unfortunately, more often this is not the case as most of the low-income countries, especially in sub-Saharan Africa, grossly under-invest in health research, particularly on capacity development and depend on foreign aid. In many countries this aid may comprise more than 90% of the health research budget [2]. This unfortunately precludes researchers and policy makers from owning and driving their own national research agenda according to their needs and wishes. Moreover, this situation is neither uniform nor being equally addressed in all countries; with some more acutely affected than others. For example, within sub-Saharan Africa there is a great disparity in both local investment on health and in receiving foreign aid for health, often with conflict-affected fragile states that need more support getting the least [3]. This is a paradox, since it is well known that, because of their inadequate capacities, low and medium conflict-affected countries not only rely heavily on international aid [4], but also have worse development indicators compared to their non-conflict affected counterparts [4,5].

Many developing countries generally lack broad-based research and scientific leadership to compete for research grants to initiate and manage research projects. Such lack of capacity greatly handicaps scientists from these regions to be in driving seats and own research programmes. This is a great disadvantage since it prevents those in malaria-endemic countries to truly set the agenda and help them solve their own problems. To be competitive, researchers from developing countries require capacity support in project writing, grant application and research project management skills. This should include training of the supporting staff in contract negotiations and in intellectual property rights management. Very often than not researchers and research managers in developing countries relinquish this to their collaborators in the north.

## Product discovery and early phase research & development

Another notable capacity deficiency in most low- and middle-income countries is lack of ability to participate in product discovery and early research and development. This includes basic research to discover new medicinal and biological compounds such as diagnostics, drugs and vaccines. Invariably most of all the current early drug and vaccine development prior to the first in man development phases (including those for malaria) takes place in the north. This stems from lack of personnel, sound regulatory framework and appropriate infrastructure including laboratory and good manufacturing practice facilities. Ironically, it is the same lack of such basic framework that undermines the development of the capacity that would free low-income countries from this situation. The lack of capacity to take part in early research and development of new products also translates into lack of pharmaceutical manufacturing facilities. It is hoped that the recently launched African Network for Drugs and Diagnostics Innovation (ANDI) whose mission is to promote sustainable product innovation to address African needs will mitigate this deficiency and stimulate drug discovery and development in sub-Saharan Africa. ANDI aims to achieve this by establishing and coordinating the formation of collaborative projects of African networks for research and development from product discovery to manufacturing including supporting capacity building. This will include providing support through direct funding, infrastructure strengthening, advocacy and fostering of public-private partnerships [6].

The dearth of capacity and activities in drug discovery and early development in developing countries is a mirrored in vaccine development. For example, the current norm in the malaria vaccine research and development pathway is to conduct all initial studies including first in man safety and immunogenicity studies (phase Ia clinical trials) in the north. This also applies for challenge model studies (phase IIa), which are now generally becoming a prerequisite for down selection of candidate vaccines to help in accelerating their development. The current costly and time consuming approach of conducting initial clinical trials in developed countries prior to moving on to endemic countries is partly due to lack of infrastructure, personnel and regulatory capacity to conduct and oversee such studies in endemic countries. To reduce cost and accelerate the development of malaria vaccine, it has been suggested that these earlier studies should also be conducted in malaria-endemic countries [7]. To overcome these constraints there is a need to strengthen broad reaching capacities including molecular biology, immunology and parasitology laboratory facilities, state of the art mosquito insectaries and competent ethical and regulatory framework.

There are also some important capacity gaps in the downstream end of research and development of products. These include project management and governance skills; data management including its ownership and utilization; and processing of vital laboratory assays. However, lack of capacity often relegates institutions from endemic countries that participate in multicentre studies to play peripheral roles, such as sample collection and processing, conducting of marginal laboratory tests and shipping samples abroad for more the more vital tests. These capacity gaps can however, be tacked with targeted capacity building. A good model that addresses this issue in malaria vaccine evaluation is the Afro-Immuno Assay (AIA) network. The network comprising of institutions from Burkina Faso, Gabon, Ghana, Senegal, Tanzania and Zimbabwe in partnership with Denmark, Netherlands and France has developed standardized enzyme immunoassays, that ensure use of same reagents, protocols and statistical methods in the evaluation candidate malaria vaccines [8]. These include assays for the Glutamate-rich Protein (GLURP), the Merozoite Surface Protein 3 (MSP3), the 19-kilo Dalton region of the Merozoite Protein 1 (MSP1_19_) and the Apical Membrane Antigen 1 (AMA1), which are among the current malaria vaccine development pipeline [8-11]. This network uses standardized protocols, common pool and sourcing of reagents, harmonized project management and centralized quality assurance and governance. The network also facilitates south-south mentoring, staff exchange and creation of a critical mass of scientists working together for a common goal. Such programmes that are owned and led by scientists from developing countries must be encouraged and emulated.

## Malaria control, elimination and eradication

Since the announcement by Bill & Melinda Gate Foundation that malaria eradication is one of their goals [12] and the endorsement of the World Health Organization (WHO) and the Roll Back Malaria (RBM) Partnership on the elimination of malaria in countries where this is possible, there has been a rekindled interest in malaria control and elimination programmes. It is generally agreed that even with the currently available tools, national or regional elimination of malaria is probably possible, especially where malaria endemicity is low [13,14]. However, in areas of high transmission regions this will require increased efforts such as scaling up of interventions and use of innovative control measures. This may include progressive containment starting with elimination in countries bordering high transmission areas and gradually moving to eliminate the disease in high endemic regions [15]. However, for this to be achieved human resource and infrastructure capacity with require to be strengthened. Skills and empowerment in data analysis, modelling and decision making will have to be improved and proliferated to support innovative control methods.

One of the innovative ways of strengthening malaria control and elimination is by the integrated vector management control. This comprises use of evidence-based decision-making; integration of different control measures; sector-wide collaboration within and outside the health sector; advocacy, social mobilization and legislation; and capacity strengthening to manage such programmes. Such capacity building should include the enabling of appropriate utilization of data in decision-making, and evaluation of the currently ongoing vector control programmes, such as use of insecticide-treated bed nets and indoor residual spraying; employment of additional vector control tools and other interventions such as case detection and treatment strategies; monitoring and evaluation of these strategies; and a continued global search and evaluation of new tools [16]. This will also require the development of essential physical infrastructure, financial resources and human capacity, at both local and national levels. One of the success stories emanating from this approach is that of the Zambia National Malaria Control Programme, which has expanded its vector control coverage and leveraged resources to develop a national capacity that has greatly improved malaria control in the country [17]. Similar success has been reported elsewhere in Africa [18]. It is therefore now very important as we come towards the “end game” and push for global and comprehensive malaria control, elimination and eventual eradication, due attention must be paid to the strengthening of the necessary capacity that is required to realize this important goal.

## Innovative ways of capacity development

In order to address some of these issues, it is encouraging to note that in recent times several international development partners and research funders have been adopting different innovative mechanisms for supporting capacity development. For instance in response to the regional disparity in capacity for health research and investment in health research in sub-Saharan Africa, the European & Developing Countries Clinical Trials partnership (EDCTP) has launched Regional Networks of Excellence for conducting clinical trials [19,20]. This scheme supports regional networks comprising various institutions of different strengths and weaknesses to collaborate in conducting clinical trials and research while supporting each other taking advantage of their strengths. This regional approach and partnering of institutions of varying strength, mitigates the negative effects of the traditional tendencies of supporting stronger institutions at the expense of the weaker ones. Other examples of innovative approaches towards capacity development include the Wellcome Trust African Institutions Initiative [21] and the recently launched Medical Research Council/ United Kingdom’s Department for International Development (MRC/DfID) African Research Leadership Scheme. It is also very encouraging to note that many of these schemes have partnerships as the foundation of their programmes [19-23] and indeed some such as the EDCTP programme encourage ownership and leadership from partners in the south, which includes mutual decision-making in prioritization of the research agenda and capacity building activities [19,20,22]. However, if any of these measures are to be effective and sustainable they will require local commitment, ownership and investment. Thus, effective capacity building must be a shared responsibility that requires proactive involvement of both international development partners and partners from the south including from private sector. Since it is the developing countries that are going to benefit most from the developed capacity in their nations, it is appropriate that they should put more effort in building that capacity. This calls for a specific efforts to determine what is required and make appropriate investments including nurturing of a conducive environment to utilize, retain and. sustain such capacity. It must however, be emphasized that this should always be accepted to be long-term endeavour, rather than a quick-fix adventure.

## Competing interests

The authors declare that they have no competing interests.

## References

[B1] European Court of Auditors European Commission development assistance to health services in sub-Saharan Africa 2009 http://eca.europa.eu/portal/pls/portal/docs/1/2482316.PDF (accessed Jan 14, 2009)

[B2] Strategic and business plan for ANDI African Network for Drugs and Diagnostics Innovation – 2^nd^ Stakeholders meeting 2009 Cape Town

[B3] Patel P Roberts B Guy S Lee-Jones L Conteh L Tracking official development assistance for reproductive health in conflict-affected countries PLoS Med 2009 6 e1000090 doi:10.1371/journal.pmed.1000090 10.1371/journal.pmed.1000090 19513098 PMC2682761

[B4] World Bank Global Monitoring Report Millennium Development Goals: confronting the challenges of gender equality and fragile states 2007 http://http://www-wds.worldbank.org/external/default/WDSContentServer/WDSP/IB/2007/04/11/000112742_20070411162802/Rendered/PDF/394730GMR02007.pdf.

[B5] O’Hare BA Southall DP First do no harm: the impact of recent armed conflict on maternal and child health in sub-Saharan Africa J R Soc Med 2007 100 564 570 10.1258/jrsm.100.12.564 18065709 PMC2121626

[B6] Mboya-Okeya T Ridley RG Nwaka S The African Network for Drugs and Diagnostics Innovation Lancet 2009 373 1207 1208 10.1016/S0140-6736(09)60111-2 19410700

[B7] Chilengi R Clinical development of malaria vaccines – should earlier trials be done in malaria endemic countries? Hum Vaccin 2009 5 9 627 636 19617708 10.4161/hv.9141

[B8] Dodoo D Aikins A Kusi KA Lamptey H Remarque E Milligan P Bosomprah S Chilengi R Osei YD Akanmoru BD Theisen M Cohort study of the association of antibody levels to AMA1, MSP1_19_, MSP3 and GLURP with protection from clinical malaria in Ghanaian children Malar J 2008 7 142 10.1186/1475-2875-7-142 18664257 PMC2529305

[B9] Lusingu JP Vestergaard LS Alifrangis M Mbando BP Theisen M Kitua AY Lemnge MM Theander TG Cytophilic antibodies to *Plasmodium falciparum* glutamate rich protein are associated with malaria protection in an area of holoendemic transmission Malar J 2005 4 48 10.1186/1475-2875-4-48 16194274 PMC1262760

[B10] Nebie I Diara A Ouedraogo A Soulama I Bougouma EC Tiona AB Konate AT Chilengi R Theisen M Dodoo D Remarque E Bosomprah S Milligan P Sirima SB Humoral responses to *Plasmodium falciparum* blood-stage antigen and association with the incidence of clinical malaria in children living in an area of seasonal malaria transmission in Burkina fas, West Africa Infect Immun 2008 76 759 766 10.1128/IAI.01147-07 18070896 PMC2223475

[B11] Sirima SB Nebie I Ouedraogo A Soulama I Cuzzin-Quattara N Cousens S Leroy O Safety and immunogenicity of the *Plasmodium falciparum* merozoite surface protein-3 long synthetic peptide (MSP3-LSP) malaria vaccine in healthy, semi-immune adult males in Burkina Faso, West Africa Vaccine 2007 25 2723 2732 10.1016/j.vaccine.2006.05.090 17280744

[B12] Roberts L Enserink M Did they really say ...eradication? Science 2007 318 1544 1545 10.1126/science.318.5856.1544 18063766

[B13] Guerra CA Gikandi PW Tatem AJ Noor AM Smith DL Hay SI Snow RW The limits and intensity of *Plasmodium* falciparum transmission: implication for malaria control and elimination worldwide PLoS Med 2008 5 e38 Doi:10.1371/journal.pmed.0050038 10.1371/journal.pmed.0050038 18303939 PMC2253602

[B14] Greenwood BM Control to elimination: implication for malaria research Trends Parasitol 2008 24 449 454 10.1016/j.pt.2008.07.002 18760671

[B15] Feachem R Sabot O A new global malaria eradication strategy Lancet 2008 371 1633 1635 10.1016/S0140-6736(08)60424-9 18374409

[B16] Beier JC Keating J Githire JI Macdonald MB Impoinvil DE Novak RJ Integrated vector management for malaria control Malar J 2008 7 Suppl 1 S4 doi:10.1186/1475-2875-S1-S4 10.1186/1475-2875-7-S1-S4 19091038 PMC2604879

[B17] Chanda KS Masaninga F Coleman M Sikaala C Katebe C Macdonals M Baboo KS Govere J Manga L Integrated vector management: the Zambian experience Malar J 2008 7 164 10.1186/1475-2875-7-164 18752658 PMC2551620

[B18] Caldas de Castro M Yamagata Y Mtasiwa D Tanner M Utzinger J Keiser J Singer BH Integrated urban malaria control: a case control study in Dar es salaam, Tanzania Am J Trop Med Hyg 2004 71 103 117 15331826

[B19] Kitua AY Corrah T Herbst K Nyirenda T Agwale S Makanga M Mgone CS Strengthening capacity, collaboration and quality of clinical research in Africa: EDCTP Networks of Excellence Tanzan J Health Res 2009 11 51 54 19445106 10.4314/thrb.v11i1.43253

[B20] Mgone CS Salami W EDCTP: a genuine north-south partnership Trop Med Int Health 2009 14 1327 1328 10.1111/j.1365-3156.2009.02391.x 19840349

[B21] The Lancet Strengthening research capacity in Africa Lancet 2009 374 1 10.1016/S0140-6736(09)61213-7 19577676

[B22] Matee MI Manyando C Ndumbe PM Corrah T Jaoko WG Kitua AY Ambene HPA Ndounga M Zijenah L Ofori-Adjei D Agwale S Shongwe S Nyirenda T Makanga M BMC Public Health 2009 9 249 doi.10.1186/1471-2458-9-249 19619283 10.1186/1471-2458-9-249 PMC2719636

[B23] Whitworth JAG Kokwaro G Kinyanjui S Snewin VA Tanner M Walport M Sewankambo N Strengthening research capacity for health in Africa Lancet 2008 372 9649 1590 1593 10.1016/S0140-6736(08)61660-8 18984193 PMC2607030

